# Brachial-Ankle Pulse Wave Velocity Reflects Regional Arterial Stiffness and Distensibility in Patients with Abdominal Aortic Aneurysm

**DOI:** 10.3400/avd.oa.24-00097

**Published:** 2025-01-01

**Authors:** Toshiya Nishibe, Shinobu Akiyama, Masaki Kano, Shoji Fukuda, Fumio Chiba, Jun Koizumi, Masayasu Nishibe

**Affiliations:** 1 Faculty of Medical Informatics, Hokkaido Information University, Ebetsu, Hokkaido, Japan; 2 Department of Cardiovascular Surgery, Tokyo Medical University, Tokyo, Japan; 3 Department of Radiology, Chiba University School of Medicine, Chiba, Chiba, Japan; 4 Department of Surgery, Eniwa Midorino Clinic, Eniwa, Hokkaido, Japan

**Keywords:** abdominal aortic aneurysm, pulse wave analysis, stiffness, distensibility

## Abstract

**Objectives:** We investigated the association between brachial-ankle pulse wave velocity (PWV) and arterial stiffness and distensibility in the aneurysmal sac of abdominal aortic aneurysm (AAA).

**Methods:** Data from 49 patients with AAA from June 2020 to November 2022 at Tokyo Medical University Hospital were retrospectively analyzed. Brachial-ankle PWV (cm/s) was obtained via an automated oscillometric method. Regional arterial stiffness and distensibility parameters, such as stiffness parameter (β), pressure-strain elasticity modulus (Ep, kPa), one-point PWV (PWV β, m/s), and arterial compliance (AC, mm^2^/kPa^−1^), were assessed using 2-dimensional automated tissue tracking (2DTT) ultrasonography. Patients were divided into two groups: high PWV (≥1800) and low PWV (<1800).

**Results:** Patients with high PWV showed significantly higher β and PWV β (30.6 ± 10.1 vs. 25.2 ± 6.3, p = 0.047; 11.6 ± 2.3 vs. 10.5 ± 1.5, p = 0.048) and significantly lower AC in the aneurysmal sac (10.6 ± 5.3 vs. 14.7 ± 8.1, p = 0.045) than those with low PWV. AC was negatively correlated with PWV (r = −0.361, p = 0.011).

**Conclusions:** Brachial-ankle PWV can reflect arterial stiffness and distensibility, as measured by 2DTT ultrasonography, in the aneurysmal sac of AAA, suggesting its potential as an elasticity index for assessing regional arterial stiffness and distensibility in AAA.

## Introduction

The pathophysiology of abdominal aortic aneurysm (AAA) includes a decrease in elastic fibers as well as an increase in collagen in the arterial wall; it is influenced by multiple factors, including acquired or genetic risk factors. These alterations in the composition of the extracellular matrix of the arterial wall result in a change in arterial stiffness or elasticity, which is an important mechanical property of the arterial system. Increased arterial stiffness or, in other words, decreased arterial elasticity, is known to play an important role in the development and progression of AAA.^1)^

A variety of parameters to evaluate arterial stiffness have been developed and introduced, including augmentation index, arterial compliance (AC), cardio-ankle vascular index, and pulse wave velocity (PWV).^2)^ Above all, PWV, which is calculated as the distance traveled by the pulse wave divided by the time taken to travel that distance, is considered the simplest and most widely used method.^3)^ Various levels were used to measure PWV, including carotid-to-femoral (carotid-femoral), carotid-to-brachial, carotid-to-radial, brachial-to-ankle (brachial-ankle), and heart-to-carotid. Arterial stiffness, as measured by brachial-ankle PWV, carotid-femoral PWV, and cardio-ankle vascular index, has been found to be higher in patients with AAA than those without, possibly reflecting increased arterial stiffness in the aneurysmal wall.^4^^–^^7)^

Brachial-ankle PWV is most often used in daily clinical practice because of its simplicity, while it has drawbacks in theoretically estimating arterial stiffness in not only AAA but also the descending thoracic aorta and/or peripheral muscular arteries; one may have concerns that brachial-ankle PWV does not reflect the elastic properties of AAA. Therefore, we examined the relationship between brachial-ankle PWV and regional arterial stiffness and distensibility parameters of the aneurysmal sac in patients with AAA. This study measured 4 regional arterial stiffness and distensibility parameters, such as stiffness parameter (β), pressure-strain elasticity modulus (Ep), one-point pulse wave velocity (PWV β), and AC, using 2-dimensional automated tissue tracking ultrasonography (2DTT; FUJIFILM Medical, Tokyo, Japan).^8^^–^^10)^ The 2DTT is based on speckle tracking which is a technique tracking natural acoustic markers, that is, speckles, present in ultrasound images. By analyzing how these speckles move over time, the 2DTT can accurately determine tissue displacement, velocity, and strain. The 2DTT is highly accurate and can analyze small, localized tissue movements in detail, localized tissue movements, which is essential for detecting subtle changes with a high resolution of 0.01 mm.

## Materials and Methods

### Patients

Data on 49 patients with AAA between June 2020 and November 2022 were obtained from a prospectively maintained database at the Department of Cardiovascular Surgery, Tokyo Medical University Hospital, and were retrospectively analyzed. At the initial consultation, all the patients were asked whether they were willing to provide written informed consent for their clinical data to be used for scientific presentations or publications. The procedures followed were in accordance with the ethical standards of the responsible committees on human experimentation (institutional and national) and the Helsinki Declaration of 1975, as revised in 2008. This study was approved by the clinical research committee of Tokyo Medical University, where it was performed (TS2020-0388, January 15, 2021).

Baseline data on demographic and clinical characteristics, including age, sex, body mass index (BMI, kg/m^2^), current smoking, past smoking, concomitant diseases (hypertension, dyslipidemia, diabetes mellitus, chronic obstructive pulmonary disease [defined as forced expiratory volume in 1 second <70%], and chronic kidney disease [defined as estimated glomerular filtration rate <60 mL/min/1.73m^2^]), history of cerebrovascular disease and ischemic heart disease, and active cancer, as well as the use of antiplatelet drugs, statins, and β-blockers, were retrieved from the database.

### Brachial-ankle PWV

Brachial-ankle PWV was measured using an automated oscillometric method (model BP-203RPEIII; Omron Colin, Tokyo, Japan) at our vascular laboratory. The subject was laid in the supine position, with electrocardiogram electrodes placed on both wrists, a microphone on the left edge of the sternum, and cuffs wrapped on both the upper arms and ankles. Pulse volume waveforms at the upper arm and ankle were recorded using a semiconductor pressure sensor (the sample acquisition frequency for PWV was set at 1200 Hz). PWV was measured after a 5-minute rest. The higher value of PWV was used in the analysis as a representative value for evaluation.

According to the physiological diagnosis criteria for vascular failure committee,^11)^ these patients were categorized into two groups: patients with high PWV (increased arterial stiffness, ≥1800 cm/s) and those with low PWV (normal or borderline arterial stiffness, <1800 cm/s). This cutoff value is applicable for the assessment of the cardiovascular risk^11)^ as well as the prediction of aneurysmal sac shrinkage after endovascular aortic repair (EVAR) for AAA.^12)^

### Arterial stiffness and distensibility parameters

Regional arterial stiffness and distensibility were evaluated with the use of 2DTT ultrasonography in a standard manner (probe of 3.5MHz, ALOKA LISENDO 880LE; FUJIFILM Medical).^8^^–^^10)^ Short-axis images of the aneurysm were digitally captured at the maximum short-axis diameter. Two points on the anterior and posterior walls of the aneurysm were manually placed using 2D images. The movement of these points was tracked during changes in systolic and diastolic blood pressure. The change in the diameter of the aneurysm was measured using the 2DTT technique. The calculated smallest detectable movement was 0.01 mm.

Regional arterial stiffness or distensibility parameters, such as β, Ep, PWV β, and AC, were calculated using the following formulae^13)^:

β = ln (Ps/Pd)/[(Ds – Dd)/Dd], where ln is the natural logarithm, unitless

Ep = (Ps–Pd)/[(Ds–Dd)/Dd], units: kPa

PWV β = 1/4 √(βPd/2ρ), units: m/s

AC = π(Ds × Ds – Dd × Dd)/[4×(Ps – Pd)], units: mm^2^ /kPa^−1^

where Ps and Pd are brachial systolic and diastolic arterial pressure, respectively, Ds and Dd are systolic and diastolic aneurysm diameter, respectively, and ρ is blood density (1050 kg/m^−3^).

### Statistics

The data are presented as the mean ± standard deviation for continuous variables. The statistical software packages PRISM 9 for MAC OS X (GraphPad Software, La Jolla, CA, USA) and Mac Tokeikaiseki Ver.3.0 (Esumi, Tokyo, Japan) were used for statistical analysis. For continuous variables, the normality of the distribution was determined using the Kolmogorov–Smirnov test. Differences in continuous variables were made between the two groups using the Student’s t-test for normally distributed data and the Mann–Whitney U test for other data. Dichotomous variables were compared between two groups using the Chi-square test or Fisher’s PLSD test, as appropriate. Correlation coefficients were obtained using Pearson regression analysis.

## Results

### Patient characteristics and comparison between patients with high and low PWV

There were 36 males (73.5%) and 13 females (26.5%), with a median age of 76 years (range 57–93 years). Table 1 compares patient characteristics, risk factors, brachial blood pressure, and ultrasonographic measurements between the two groups: patients with high PWV (n = 26) and those with low PWV (n = 23). No significant differences were found in patient characteristics, risk factors, brachial blood pressure, or ultrasonographic measurements between the two groups.

**Table:** Table 1 Patient characteristics and comparison between patients with low and high PWV

Variables	Total (n = 49)	Low PWV (n = 26)	High PWV (n = 23)	*p* values
Age	76.9 ± 8.5	74.9 ± 8.7	79.2 ± 8.5	0.087
>75 years	30 (61.6)	14 (53.8)	16 (69.6)	0.260
Female gender	13 (26.5)	7 (26.9)	6 (26.1)	0.066
Body mass index		21.8 ± 3.6	21.9 ± 3.6	0.923
>25 kg/m^2^	9 (18.4)	4 (15.4)	5 (21.7)	0.717
Smoking				
Current	17 (34.7)	11 (42.3)	6 (26.1)	0.234
Past	33 (67.3)	19 (73.1)	14 (60.9)	0.363
Anatomy				
Aneurysm diameter (mm)	51.0 ± 8.3	51.6 ± 8.3	50.4 ± 8.3	0.588
Small aneurysm (<50 mm)	36 (73.5)	20 (76.9)	16 (69.6)	0.560
Mural thrombus	34 (28.6)	17 (65.4)	17 (73.9)	0.250
Concomitant diseases				
Hypertension	36 (73.5)	17 (65.4)	19 (82.6)	0.702
Dyslipidemia	14 (28.6)	9 (34.6)	5 (21.7)	0.360
Diabetes mellitus	9 (18.4)	7 (26.9)	2 (8.7)	0.154
Cerebrovascular disease	10 (20.4)	4 (15.4)	6 (26.1)	0.483
Ischemic heart disease	18 (24.5)	9 (34.6)	9 (21.7)	0.744
Chronic obstructive lung disease	12 (24.5)	6 (23.1)	6 (26.1)	0.807
Chronic kidney disease	17 (34.7)	12 (46.2)	5 (21.7)	0.132
Medication				
Antiplatelet drugs	22 (44.9)	15 (57.7)	7 (30.4)	0.056
Statins	24 (49.0)	14 (53.8)	10 (43.5)	0.469
β-blockers	19 (38.8)	12 (46.2)	7 (30.4)	0.260
Brachial blood pressure (mmHg)				
Systemic (Ps)	120 ± 14	119 ± 15	122 ± 14	0.557
Diastolic (Pd)	71 ± 10	69 ± 11	72 ± 9	0.325
Mean	87 ± 10	86 ± 10	89 ± 9	0.298
Ultrasonographic measurements (mm)				
Systolic (Ds)	49.11 ± 8.67	50.66 ± 9.00	47.36 ± 5.13	0.222
Diastolic (Dd)	50.20 ± 8.49	51.92 ± 8.82	48.25 ± 7.84	0.180
PWV	1899 ± 523	1560 ± 162	2283 ± 527	<0.001*

Unless indicated otherwise, data are presented as mean ± SD or n (%); *Significant.

PWV: pulse wave velocity

### Comparison in regional arterial stiffness and distensibility parameters between patients with high and low PWV

**Figure 1:**
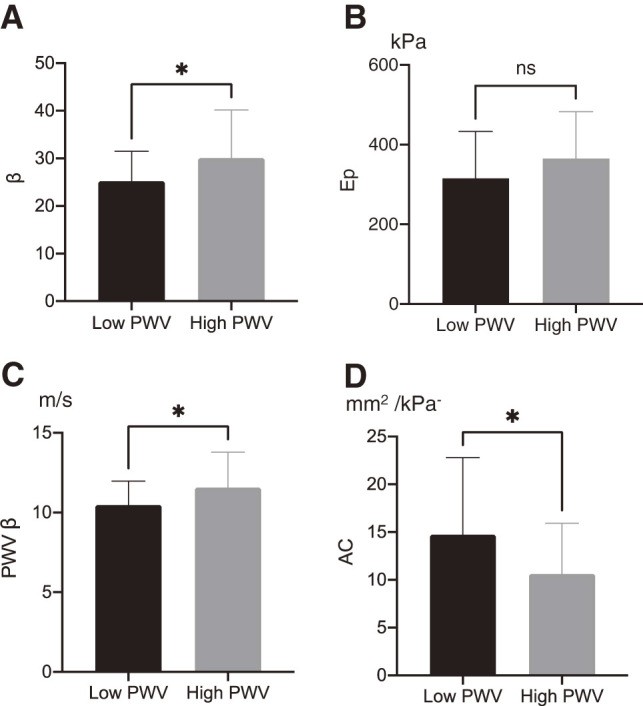
Fig. 1 Comparison in regional arterial stiffness and distensibility parameters between patients with high and low PWV. *Significant. (**A**) Stiffness parameter (β). (**B**) Pressure-strain elasticity modulus (Ep). (**C**) One-point pulse wave velocity (PWV β). (**D**) Arterial compliance. PWV: pulse wave velocity

There were no significant differences in systolic, diastolic, and mean pressures between the two groups (Table 1). Figure 1 shows that β and PWV β are significantly higher in patients with high PWV compared to those with low PWV (30.6 ± 10.1 vs. 25.2 ± 6.3, *p* = 0.047; 11.6 ± 2.3 vs. 10.5 ± 1.5, *p* = 0.048, respectively), while AC is significantly lower (10.6 ± 5.3 vs. 14.7 ± 8.1, *p* = 0.045); although not reaching statistical significance, Ep tends to be higher in patients with high PWV compared to those with low PWV (365.1 ± 117.6 vs. 315.4 ± 117.7, *p* = 0.079).

### Correlation between brachial-ankle PWV and regional arterial stiffness and distensibility parameters

**Figure 2:**
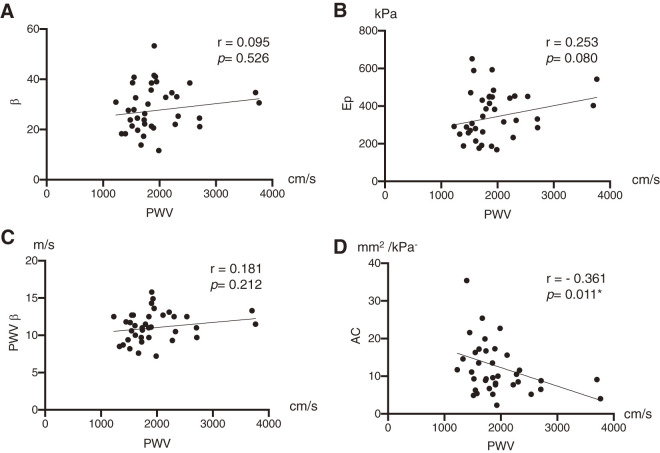
Fig. 2 Scatterplots demonstrating correlations of PWV to regional arterial stiffness and distensibility parameters. *Significant. (A) Stiffness parameter (β). (B) Pressure-strain elasticity modulus (Ep). (C) One-point pulse wave velocity (PWV β). (D) Arterial compliance. PWV: pulse wave velocity

Pearson regression analyses were used to determine the correlations between brachial-ankle PWV and regional arterial stiffness or distensibility parameters. Figure 2 shows that AC is significantly negatively correlated with brachial-ankle PWV (r = −0.361, *p* = 0.011), whereas there are no significant correlations between brachial-ankle PWV and β, Ep, or PWV β (r = 0.095, *p* = 0.526; r = 0.253, *p* = 0.080; r = 0.181, *p* = 0.212, respectively).

## Discussion

This study shows a significant association between high brachial-ankle PWV, defined as PWV ≥1800 cm/s, and regional arterial stiffness and distensibility parameters, specifically β, AC, and PWV β, in patients with AAA; both β and PWV β were significantly higher in patients with high PWV compared to those with low PWV, while AC was significantly lower. Additionally, AC showed a significant negative correlation with brachial-ankle PWV. These results suggest that a high brachial-ankle PWV may reflect increased arterial stiffness, indicated by β and PWV β, and decreased arterial distensibility, as represented by AC, in the aneurysm sac of AAA.

Regional arterial stiffness and distensibility in AAA were assessed in this study using 2DTT ultrasonography.^8^^–^^10^^)^ The 2DTT technique can capture high-frequency signals generated during the systolic and diastolic motion of the proximal and distal arterial wall, allowing for the manual calculation of mechanical variables that reflect arterial wall characteristics, including β, Ep, PWV β, and AC.^13)^ Here, β represents arterial wall stiffness independent of blood pressure, Ep indicates an artery’s resistance to deformation, PWV β is derived from β to provide information on arterial stiffness at a specific region of interest, and AC reflects the absolute change in arterial diameter or area for a given pressure change. The 2DTT has proven to be a valid, reliable, and reproducible method for characterizing blood vessels.^9)^

The mechanical properties of the abdominal aorta are primally determined by the matrix components of the wall, including elastin, collagen, and smooth muscle cells.^14)^ Consequently, changes in the composition and structure of the aneurysmal wall alter its mechanical properties; elastin degradation or reduction results in loss of elasticity, while collagen production or an increase in collagen content can elevate wall stiffness. Previous studies using an echo-tracking imaging system demonstrated that Ep and β of the aneurysmal wall were elevated in individuals with AAA compared to those without, whereas AC was decreased.^15^^,^^16)^ Additionally, arterial stiffness, expressed as β, may be related to clinical outcomes, such as future growth rate and risk of rupture.^17^^,^^18)^

The basic assumption in calculating PWV is that the artery functions as a mechanically and geometrically homogeneous straight duct. Theoretically, changes in arterial geometry influence regional hemodynamics, while alterations in wall properties are expected within the aneurysmal segment of the aorta. However, previous studies have found no association between the size of the aneurysm and PWV;^5^^,^^19)^ a reduction in lumen size due to luminal thrombus formation may counteract the effect of aneurysmal dilation on regional pulse wave propagation, whereas patients with AAA had higher aortic wall stiffness within the aneurysm,^20)^ potentially affecting pulse wave propagation.^21)^ This study also revealed significant differences in regional arterial stiffness and distensibility parameters, such as β, PWV β, and AC, but not aneurysm diameter, above and below the brachial-ankle PWV, defined with as a threshold of 1800 cm/s.^11^^,^^12)^

Recently, brachial-ankle PWV has been utilized to predict aneurysm sac behavior after EVAR.^12^^,^^22^^,^^23)^ Low preoperative PWV, defined as < approximately 1800 cm/s, has been associated with aneurysm sac shrinkage after EVAR, suggesting that arterial stiffness or elasticity is one of the key factors influencing sac behavior postoperatively.^12^^,^^23)^ This study indicates that brachial-ankle PWV may reflect the mechanical properties of the aneurysmal wall and could help predict aneurysmal sac behavior after EVAR for AAA.

As shown in this study, AC was significantly negatively correlated with brachial-ankle PWV, whereas there were no significant correlations between brachial-ankle PWV and β, Ep, or PWV β. These data suggest that aneurysmal wall stiffness, as indicated by β, Ep, and PWV β, may vary less even than aneurysmal wall distensibility, expressed as AC, across AAAs. However, arterial wall stiffness and distensibility were only assessed at the maximum anteroposterior diameter in this study, and the visual presentation of results did not provide a spatial view of the AAA over the entire recorded sequence.^24)^ Further studies are needed to analyze the spatially resolved strain field of the aneurysmal wall using 3D ultrasound or 4D flow magnetic resonance imaging.

### Study limitations

Some limitations should be acknowledged. First, the study is based on a single-center database with a relatively small sample size. In a smaller cohort, individual variations in patient characteristics and arterial properties may have a larger impact on the results, potentially obscuring the true relationship between each parameter and PWV. Second, peripheral blood pressure measurements are known to overestimate central blood pressure, especially in young populations, and brachial blood pressure was used to calculate regional arterial stiffness and distensibility parameters. Finally, brachial blood pressure measurements were taken immediately before starting the examination, assuming that hemodynamic parameters did not change significantly during the examination.

## Conclusion

Brachial-ankle PWV can reflect arterial stiffness and distensibility, as measured by 2DTT ultrasonography, in the aneurysmal sac of the AAA. This finding provides novel insights into the potential of brachial-ankle PWV as an elasticity index for assessing regional arterial stiffness and distensibility of AAA.

## Declarations

### Funding

Toshiya Nishibe received KAKENHI (grant no. 22K08926) from the Japan Society for the Promotion of Science (JSPS).

### Disclosure statement

The authors declare that they have no conflicts of interest.

### Author contributions

Study conception: TN

Data collection: TN, MK, SA, and SF

Analysis: TN, MN, and JK

Investigation: TN, MK, and SA

Manuscript preparation: TN and FC

Funding acquisition: TN

Critical review and revision: all authors

Final approval of the article: all authors.
